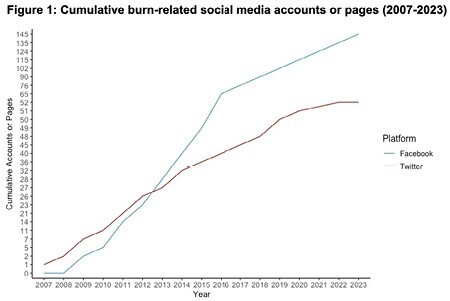# 109 Practice Promotion or Burn Prevention: An Assessment of the Social Media Landscape in Burn Care

**DOI:** 10.1093/jbcr/irae036.108

**Published:** 2024-04-17

**Authors:** Artur Manasyan, Erin E Ross, Nicolas Malkoff, Brigette Cannata, Haig A Yenikomshian, Justin Gillenwater

**Affiliations:** Keck School of Medicine, University of Southern California, Los Angeles, CA; Keck School of Medicine of USC, Carlsbad, CA; Keck School of Medicine of USC, Staten Island, NY; University of Southern California, Los Angeles, CA; Keck Medicine of USC, Los Angeles, CA; Keck School of Medicine, University of Southern California, Los Angeles, CA; Keck School of Medicine of USC, Carlsbad, CA; Keck School of Medicine of USC, Staten Island, NY; University of Southern California, Los Angeles, CA; Keck Medicine of USC, Los Angeles, CA; Keck School of Medicine, University of Southern California, Los Angeles, CA; Keck School of Medicine of USC, Carlsbad, CA; Keck School of Medicine of USC, Staten Island, NY; University of Southern California, Los Angeles, CA; Keck Medicine of USC, Los Angeles, CA; Keck School of Medicine, University of Southern California, Los Angeles, CA; Keck School of Medicine of USC, Carlsbad, CA; Keck School of Medicine of USC, Staten Island, NY; University of Southern California, Los Angeles, CA; Keck Medicine of USC, Los Angeles, CA; Keck School of Medicine, University of Southern California, Los Angeles, CA; Keck School of Medicine of USC, Carlsbad, CA; Keck School of Medicine of USC, Staten Island, NY; University of Southern California, Los Angeles, CA; Keck Medicine of USC, Los Angeles, CA; Keck School of Medicine, University of Southern California, Los Angeles, CA; Keck School of Medicine of USC, Carlsbad, CA; Keck School of Medicine of USC, Staten Island, NY; University of Southern California, Los Angeles, CA; Keck Medicine of USC, Los Angeles, CA

## Abstract

**Introduction:**

With the recent surge in social media use, the public has become increasingly reliant on online content for information. Social media offers a readily available, cost-effective way for medical experts to disseminate knowledge and shape public health outcomes but also allows for the spread of misinformation. Given that social media is being more commonly employed as a source of medical information, this study aims to present the current landscape of burn-related content on social media. We seek to characterize the types of organizations and individuals who are publishing burn-related content, categorize the type of content produced, and describe content and creator output on popular social media platforms.

**Methods:**

Facebook, TikTok, and X (formerly Twitter) were queried with the following search terms: “burn,” “burn injury” and “burn treatment.” Identified accounts were then manually screened for relevance. Year of creation and engagement metrics were collected. Accounts were categorized by content type (education, prevention, advocacy, support, medical services promotion, product promotion, or personal experience) and creator (academic, nonprofit, medical provider, medical center, support group, for-profit business, or personal account). Data is reported using descriptive statistics and visualized graphically to explore trends.

**Results:**

Our search yielded 408 profiles, 259 of which met the inclusion criteria. The year of account creation ranged from 2007-2023, with the greatest number of new accounts created in 2016 (10.4%) (Fig. 1). TikTok had the most engagement at a median of 43,500 followers per account, with 39.0% of accounts focusing on individual experiences of burn survivors primarily on personal accounts (47.5%). In contrast, content on Facebook was related to the promotion of medical services (39.0%), where the most represented creator type was medical centers (34.2%). Nonprofits made up 36.5% of accounts on X and more than half of the content focused on patient advocacy, support, or burn prevention (51.9%) (Fig. 2).

**Conclusions:**

Medical centers and nonprofits recognize the power of social media in promoting their services for burn care and advocating for burn patients. The video-based TikTok platform attracted more informal content focusing on the personal experiences of those afflicted with burn injury. In recent years, X has become a less popular medium, likely due to the advent of TikTok, which can be the target of future research to further improve engagement with burn content. Given the relative lack of content focusing on burn education and prevention, we encourage a greater presence of medical professionals and organizations with expertise in burn care on popular social media platforms to fill this content gap.

**Applicability of Research to Practice:**

The findings of this study can be considered by burn professionals in their decision to engage in content creation and in determining which platform is most suitable for their content.